# Asymmetry and Asynchrony in Postnatal Growth Patterns of Two Sympatric Species of Antarctic Penguins

**DOI:** 10.1002/ece3.73563

**Published:** 2026-05-14

**Authors:** Min‐Su Jeong, Hankyu Kim, Woo‐Shin Lee, Chang‐Yong Choi

**Affiliations:** ^1^ Korea Institute of Ornithology Kyung Hee University Seoul Republic of Korea; ^2^ Department of Biology Kyung Hee University Seoul Republic of Korea; ^3^ Department of Agriculture, Forestry, and Bioresources Seoul National University Seoul Republic of Korea; ^4^ Research Institute for Agriculture and Life Sciences Seoul National University Seoul Republic of Korea

**Keywords:** adaptive‐growth hypothesis, avian growth models, breeding ecology, hatching order, morphometrics, postnatal development

## Abstract

Differential hatching timing among siblings may result in hierarchical resource distribution, leading to disadvantages for later‐hatched nestlings. Later‐hatched chicks may need to invest more in developing their skeletal structures that are related to locomotion to catch up with first‐hatched chicks and better survive after leaving the nest, resulting in greater fitness for the breeding pair. We analyzed chick growth patterns during the guarding period according to hatching order for Gentoo ( *Pygoscelis papua* ) and Chinstrap ( *P. antarcticus* ) Penguins at Narebski Point, King George Island, in Antarctica. We fitted the four‐parameter Unified‐Richards growth curve to chick measurement data. For Gentoo Penguins, growth curves showed that first chicks reached a maximum growth rate earlier than did second chicks in all body traits, leading to the growth rate of the second chicks overtaking that of first chicks from mid or late guarding period. The maximum growth rate for second chicks of Chinstrap Penguins was achieved later than that for the first chicks, only in body mass. Our results indicate that developmental strategies could differ between sympatric species with a close phylogenetic relationship, and that second chicks prioritize energy allocation to reduce asymmetry of skeletal traits during the early life stages. For field applications, our growth models provided reliable age estimates regardless of hatching order. Flipper length was the most accurate estimator for Gentoo Penguin chicks, while body mass and total head length were more reliable for Chinstrap Penguins.

## Graphical abstract

Two sympatric penguin species breeding at Narebski Point, King George Island in West Antarctica, show differences in growth asymmetry between siblings, indicating differences in early life history strategies. Chinstrap Penguin chicks grow less asynchronously, while Gentoo Penguin chicks have stronger asynchrony, suggesting Gentoo Penguins may have more flexible growth strategies than their sympatric counterpart.

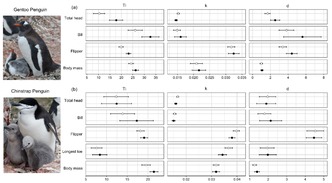

## 1 Introduction

Postnatal growth is a fundamental life‐history trait closely related to individual fitness in species with determinate growth (Starck and Ricklefs 1998). Birds are typical taxa with determinate growth, in which most individual development and growth are finished before fledging from the nest. Understanding postnatal growth and development of these species can provide valuable information on how experiences during the early life stage can affect population demographics, such as survival, fecundity, and thus individual fitness (English et al. 2016; Lindström 1999). The life history during early developmental periods can be used to inform species conservation and management efforts (Cam and Aubry 2011).

In long‐lived seabirds, morphometric traits and body conditions during postnatal development of pre‐fledging chicks are often related to post‐fledging survival, recruitment, and age at first return to natal colonies (Maness and Anderson 2013; Monticelli and Ramos 2012; Morrison et al. 2009). While growth rates widely vary among species according to life‐history strategy (Ricklefs 1968a; Starck and Ricklefs 1998), intra‐species variation can be influenced by environmental conditions (Blanco et al. 1996; Gebhardt‐Henrich and Richner 1998; Sauve et al. 2023).

In species that lay more than one egg per clutch, different hatching timing between chicks within a clutch (hatching asynchrony) can lead to a hierarchical distribution of food and other resources with respect to hatching order (Blanco et al. 1996; Robertson et al. 2015). Chicks that hatch early may also have a competitive advantage in begging and attracting parents' attention (Drummond 2002; Royle et al. 2002; van Heezik and Seddon 1991). Such hierarchical food distribution results in different growth rates and size asymmetry among siblings, which can cause higher mortality rates among later‐hatched chicks when prey availability is low (Merkling et al. 2014; Stienen and Brenninkmeijer 2006; van Heezik and Seddon 1991).

However, some species balance biased growth with hatching asynchrony. For example, in African Penguins ( *Spheniscus demersus* ) and Chinstrap Penguins ( *Pygoscelis antarctica* ), asymmetry in chicks' flipper length and body mass between siblings during the early postnatal rearing period reportedly decreased as the breeding season progressed (when measured on the same day) (Moreno et al. 1994; van Heezik and Seddon 1996). Later‐hatched chicks may increase their growth rate after the first‐born chicks, to reduce competition and result in increased fitness of the breeding pair (van Heezik and Seddon 1996).

Gentoo ( *Pygoscelis papua* ) and Chinstrap Penguins ( *P. antarcticus* ) are long‐lived seabirds that lay two eggs at intervals of three to four days (Lishman 1985; Williams 1980). This interval in egg‐laying within a clutch results in hatching asynchrony of chicks within a brood (mean asynchrony: 1.0 days in Chinstrap Penguins from Moreno et al. 1994; 1.6 days in Gentoo Penguins from Lamey 1990). The semi‐precocial chicks are brooded and guarded by a parent until approximately 30 days after hatching (hereafter “guarding period”). After this period, both parents begin to forage simultaneously as the food requirements of chicks increase, and chicks fledge from nests and form crèches (Viñuela et al. 1996). A previous study found that initial differences in body mass disappeared near the fledgling period in Chinstrap Penguins (Moreno et al. 1994). Volkman and Trivelpiece (1980) compared the average growth rates of the first and second chicks of penguins in the *Pygosceli*s genus by using coefficients of linear regression for size and mass as a function of age, but found no intraspecific differences in the growth rates of the three species (Gentoo, Chinstrap, and Adélie Penguins *P. adeliae* ). However, the non‐linear nature of chick growth makes it difficult to express changes in growth over time when the average growth is represented as a fixed slope in a linear function.

Volkman and Trivelpiece (1980) also observed variation in growth rates among body parts in pygoscelid penguins. Such differential postnatal development of body parts can be explained by an adaptive growth (development) hypothesis, in which chicks allocate nutrients to the growth of organs and body parts that are more critical to survival during early life stages; that is, they may prioritize the development of hindlimbs that could be essential for feeding chases on land later in the later chick‐rearing period (Bustamante et al. 1992; O'Connor 1977; Gownaris and Boersma 2021; Volkman and Trivelpiece 1980). This stage is followed by increasing needs for mobility in aquatic environments, where they need to chase prey and outrun predators; energy is therefore diverted to flipper growth. Adaptive growth is a beneficial trait for a chick's fitness, and we expected that asymmetry in body size would be reduced for body parts related to locomotion on land (chasing, feet) and then in the aquatic environment (flippers and bill for foraging).

Although Gentoo and Chinstrap Penguins are in the same genus, using similar food resources and often breeding at the same location, the two species are adapted to different life history traits. Chinstrap Penguins are long‐distance migrants and arrive later at the breeding colony compared with Gentoo Penguins in the same breeding colony (Wilson et al. 1998; Hinke et al. 2015), and thus may have a much more limited breeding window in the short Antarctic summer. This can lead to differential adaptation in postnatal growth strategies between these two species. Migratory species are often more time‐constrained than their resident counterparts, as they need sufficient time to raise their chicks, undergo post‐breeding body molt, and prepare for autumn migration (Alves et al. 2013). Such temporal constraints can drive differential adaptation in postnatal growth strategies between species (Reed and Clark 2016). Late‐hatching or time‐constrained chicks have been shown to exhibit accelerated growth rates, particularly for morphological traits critical to fledging, such as wing development (motor function) in Rhinoceros Auklets *Cerorhinca monocerata* (Hirose et al. 2012). This suggests that Chinstrap Penguins, with their more rigid breeding phenology, may face stronger selective pressure for rapid chick growth (time‐minimization) compared with Gentoo Penguins, which can acclimate to environmental conditions that allow for a more flexible breeding window and thus invest in each chick separately (risk‐spreading). Additionally, species‐specific responses to environmental cues—with Chinstraps being more sensitive to food productivity and Gentoos responding to temperature conditions (Juarez Martínez et al. 2026) may further shape differential growth investment strategies between siblings and between species.

In addition to these theoretical predictions, the growth curves of natal chicks can be used to estimate the age of birds in the field. For example, we can determine their hatching dates from a single measurement, rather than relying on frequent, repeated nest‐visit surveys. This method can be particularly efficient for monitoring seabirds that breed on remote islands, where researcher visits to determine hatching dates may be limited (Jeong et al. 2021; Ricklefs and White 1975; Villegas et al. 2013). The precision and accuracy of age estimates can vary by the choice of morphological measurement, hatching order, and chick age, due to differential growth rates along the sigmoidal curve and body parts (Lok et al. 2014). Current growth models used to study penguin chicks are based on linear growth patterns; hence, using a non‐linear growth curve model could improve age estimation.

In this study, we describe the postnatal growth pattern of multiple morphometric measurements and body mass of Gentoo and Chinstrap Penguin chicks during the guarding period using a four‐parameter Unified‐Richards model (U‐Richards model; Richards 1959; Svagelj et al. 2019; Tjørve and Tjørve 2010a, 2010b). We selected the U‐Richards model for its flexibility due to the adjustable inflection point, enabling more precise estimation of the non‐linear growth observed in these penguin species compared to the three‐parameter models (Svagelj et al. 2019; Tjørve and Tjørve 2017). First, we predicted that the body sizes of the second chicks would catch up with those of the first chicks in the later guarding period (growth asynchrony). Second, based on the adaptive‐growth hypothesis (growth asymmetry), we expected that these growth patterns would not be equal across measured body parts, as chicks would need to develop limbs that are necessary for obtaining food during the post‐crèching period. This may result in variation in the asymmetry of body measurements between the first and second chicks during the guarding period. Third, we discussed how life‐history and ecological traits of two sympatric and congeneric species can lead to differences in postnatal development strategies. In addition to testing these hypotheses, we provide formulas for estimating the age of chicks for each species for field applications and assess the accuracy of growth models for estimating chick age using different morphometrics, accounting for hatching order.

## 2 Methods

### 2.1 Study Site and Breeding Phenology of Study Species

Field data were collected at a breeding colony of Chinstrap and Gentoo Penguins at Narebski Point (62°14′ S, 58°46′ W), an Antarctic Specially Protected Area (No. 171), on King George Island in the South Shetland Islands. We measured chicks from the colony in four austral summers (December to January of 2013–14 to 2016–17; hereafter, austral summers are referred to by the year in which they commence) as part of a long‐term monitoring study of the Chinstrap and Gentoo Penguin populations. The breeding population sizes of Chinstrap and Gentoo Penguins were 2992 ± 136 (mean and standard deviation; range = 2850–3157 pairs) and 2317 ± 222 pairs (2112–2604 pairs), respectively, during the study period. During the study period, the average hatch date for Gentoo Penguins (mean day of year and standard deviation = 344 ± 7.5) was earlier and more variable than that of Chinstrap Penguins (mean day of year and standard deviation = 358 ± 3.5). Chinstrap Penguin adults guard their chicks for approximately 4 weeks (28 days), and then chicks form a crèche and leave for the ocean when they are between 53 and 57 days old (Viñuela et al. 1996). Gentoo chicks leave nests and begin to form crèche at an age of 25–30 days and depart breeding colonies at approximately 75–100 days old (population in South Georgia; Williams 1990). We therefore treated the first 30 days after hatching as the guarding period for both species.

### 2.2 Sampling Methods and Morphometric Measurements

From 2013 to 2014, we checked the hatching status of 500–1000 randomly selected nests every 3–5 days to estimate the mean hatching timing of each species in the colony in each season. Within these nests, we randomly reselected 18 nests of Chinstrap Penguins (*n* = 7 in 2013 and *n* = 11 in 2014) and 14 nests of Gentoo Penguins (*n* = 9 in 2013 and *n* = 5 in 2014) for which we were able to identify the precise hatching dates and then monitored the growth of two chicks over time (i.e., nests with eggs and chicks that hatched on the day of sampling nest selection, still wet from the egg contents; Table 1). We marked nests with numbered aluminum stakes at the edge of the nests for identification and visited them every day until both chicks had hatched. The stakes were retrieved after each field season. These nests were visited at short intervals to measure chick morphometrics (Table 1). Specifically, Chinstrap Penguin nests were checked 7.08 ± 1.25 times, ranging from 4 to 9 times, and the mean interval between visits for each nest spanned 3.00 to 8.00 days. Gentoo Penguin chicks were measured 10.96 ± 4.06 times, varying from 4 to 15 times, and the mean interval between these visits for each nest ranged from 2.38 to 6.33 days. In addition, we measured the morphometrics of Chinstrap Penguin chicks twice (at 0 to 3‐day‐old and 27 to 30‐day‐old of first‐hatching chicks) in 29 nests with confirmed hatching dates of the first chicks from 2014 to 2016 (Table 1). These chicks were randomly selected and measured for a separate study linking parental diet and chick condition.

**TABLE 1:** Summary of the number of sampled nests (*n*), mean and standard deviation (SD) of the measurement interval (days), and the number of visits for each species and year.

Species	Year	*n*	Intervals (mean ± SD)	Number of visits (mean ± SD)
Gentoo Penguin *Pygoscelis papua*	2013	9	2.67 ± 0.19	13.78 ± 1.11
	2014	5	5.48 ± 0.93	5.90 ± 1.66
Chinstrap Penguin *Pygoscelis antarcticus*	2013	7	4.27 ± 0.21	7.21 ± 0.58
	2014	11	5.07 ± 1.23	7.00 ± 1.54
		8	21.97 ± 5.19	2.31 ± 0.48
	2015	7	27.71 ± 1.27	2.00 ± 0.00
	2016	14	25.76 ± 4.29	2.11 ± 0.42

*Note:* All nests had two chicks in all survey periods.

Selected nests were approached slowly on foot, and a single chick was taken out from the nest for measurement while a parent bird was brooding the other chick and returned to the nest as the other chick was being measured. Adult Chinstrap Penguins will defend and peck any approaching objects, so we blocked their head with our hands and arms when returning chicks to prevent adults from pecking their chicks. For Chinstrap Penguins, the age of each chick at the last measurement was 28.96 ± 2.77 days old (*n* = 92), except for a nest that failed in the early brooding period. The failed nest was measured four times until the chicks were 10 and 9 days old, respectively. For Gentoo Penguins, the age at the last measurement was 31.32 ± 5.28 days (*n* = 28). If two chicks hatched on the same day, we determined their hatching order by visually assessing the presence of remaining liquid egg contents on downy feathers (chicks a few hours older were drier). Markings of different colors were applied under the flippers with a non‐toxic pet nail polish pen (Pawdicure Polish Pen, Warren London, USA) to identify individual chicks. The average hatching asynchronies for Chinstrap and Gentoo Penguins were −1.11 ± 0.76 days and −1.21 ± 0.80 days, respectively. The number of nests in which chicks hatched on the same date was eight (17%) in Chinstrap Penguins and three (21%) in Gentoo Penguins.

We measured the lengths of the total head (supraoccipital tip to bill tip), bill (the length from the feather margin to the bill tip), and longest toe (metatarso‐phalangeal joint to the third digit nail tip, including soft tissues over the joint—a typical measurement for penguins that is often referred to as “foot” though anatomically it is the length of the longest toe, its nail and soft tissue) with Vernier calipers to a precision of ±0.1 mm, and flipper lengths (from the base of the humerus to the flipper tip, measured from the underside of the flippers), with a 150 and 300 mm steel ruler, to ±1 mm. We began measuring the longest toe length in the middle of repeated measurements in the 2013 season and therefore have a limited number of measurements for this metric for each species. This restricted our ability to model and test the growth of the longest toe for Gentoo Penguins and to make age predictions for either species using the longest toe length. We weighed body mass using cotton bags, a spring scale (30, 50, 100, 300, 1000 ± 1 and 1000 ± 10 g scale; PESOLA, Switzerland), and electronic balances (50 kg ±10 g, Han Sung, Korea), depending on the mass of the chick.

### 2.3 Growth Model Fitting

**FIGURE 1:**
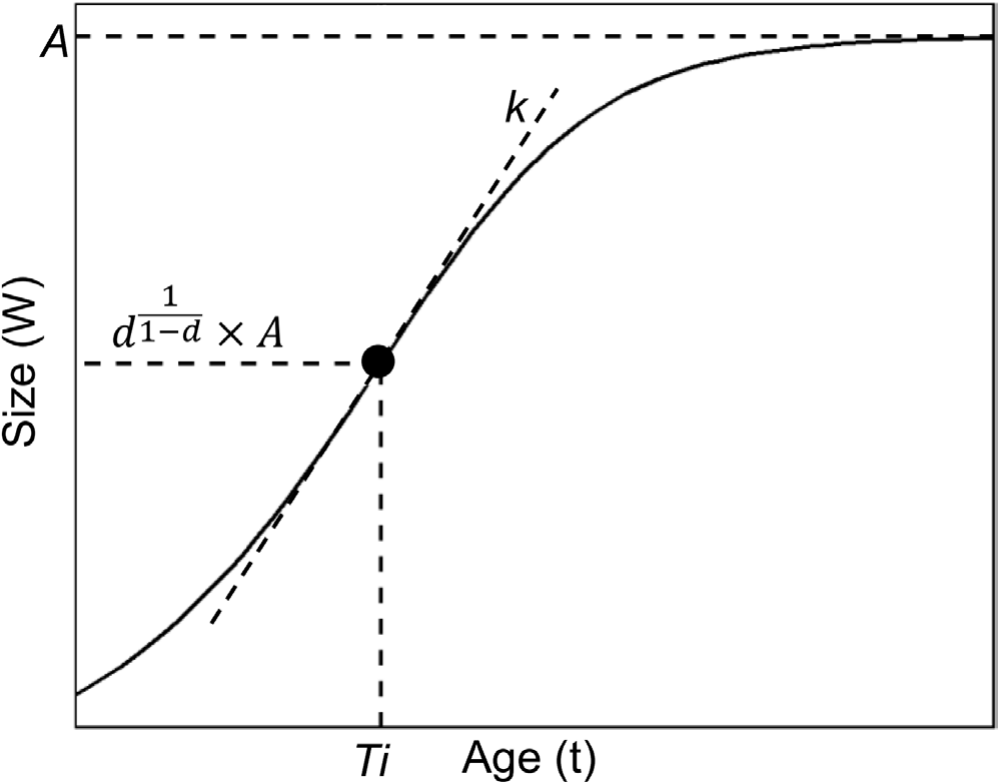
The roles of the parameters of a unified‐Richards curve in postnatal growth modeling. Parameters *A, Ti*, and *k* represent the upper asymptote of the curve, the inflection point, and the slope at the inflection point, indicating maximum body size or mass, age of maximum relative growth rate, and maximum relative growth rate, respectively. Parameter *d* determines the body size or mass at the inflection point.

Fitting a sigmoid‐shaped growth curve to empirical data from the field is useful for describing growth patterns, examining inter‐ and intraspecific variation, and estimating the age of chicks (Starck and Ricklefs 1998). Traditional growth curves commonly applied to model bird growth include logistic, Gompertz, and van Bertalanffy models; each is characterized by three parameters representing the maximum body size or mass (*A*), the age at maximum daily growth rate (*Ti*), and the maximum daily growth rate (*k*), respectively (Ricklefs 1968a, 1973; Starck and Ricklefs 1998; Tjørve and Tjørve 2010b). The age at maximum daily growth rate (inflection point of the curve) for these curves is fixed at a different proportion of the maximum body size or mass (Tjørve and Tjørve 2010a, 2010b). However, the fixed placement of the inflection point can result in unrealistic parameter estimates if the actual inflection point lies far from the fixed placement forced by the models (Tjørve and Tjørve 2017). Thus, a four‐parameter Richards model includes an extra parameter (*d*: shape) that determines the placement of the inflection point (Richards 1959; Tjørve and Tjørve 2010a). The *d* parameter allows for the inflection point to move freely along the growth curve, giving the Richards model its flexibility (Figure 1). Tjørve and Tjørve (2010b) proposed a re‐parametrized Richards model equation (Unified‐Richards or U‐Richards model) with a returning parameter for estimating actual growth rates, and the performance of the U‐Richards model has proven to be superior for semi‐precocial seabirds (Svagelj et al. 2019; Tjørve and Tjørve 2017).

We fitted a U‐Richards growth curve for each morphometric measurement and body mass of the first and second chicks using maximum likelihood methods (Pinheiro et al. 2022; Pinheiro and Bates 2000; Tjørve and Tjørve 2017). We only fitted the growth curve of the longest toe length for Chinstrap Penguins due to the small sample size. Growth curves for each species and hatching order were fitted. In the equation, *W*_*t*_ is the size or mass at age *t*. Parameters *A, Ti*, and *k* indicate the upper asymptote (maximum size or mass), inflection point (age at maximum relative growth rate), and the slope at the inflection point (maximum relative growth rate), respectively (Tjørve and Tjørve 2017; Figure 1). Parameter *k* is relative to *A* at *Ti*. Parameter *d* determines the relative size at the inflection point (at the age of maximum relative growth rate; Figure 1).

$$W_{t}=A\times {\left[1+\left(d-1\right)\times e^{\frac{-k\left(t-\mathrm{Ti}\right)}{d^{\frac{d}{1-d}}}}\right]}^{\frac{1}{1-d}}$$

**FIGURE 2:**
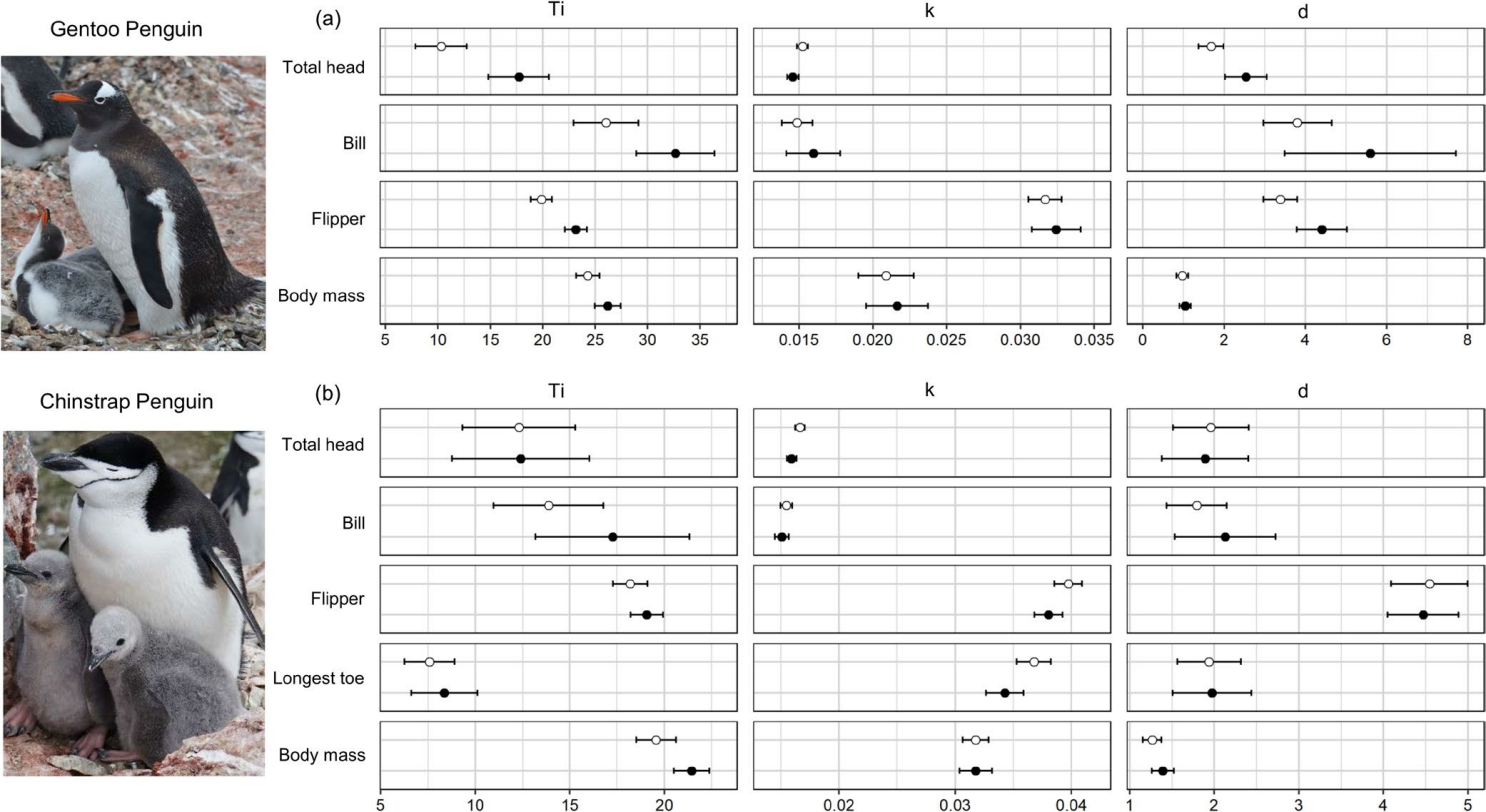
Parameter estimates (mean and 95% confidence interval) of the growth curves for morphometric measures and body mass of chicks of (a) Gentoo ( *Pygoscelis papua* ) and (b) Chinstrap Penguins ( *P. antarcticus* ). Open and filled circles represent first and second chicks, respectively.

**FIGURE 3:**
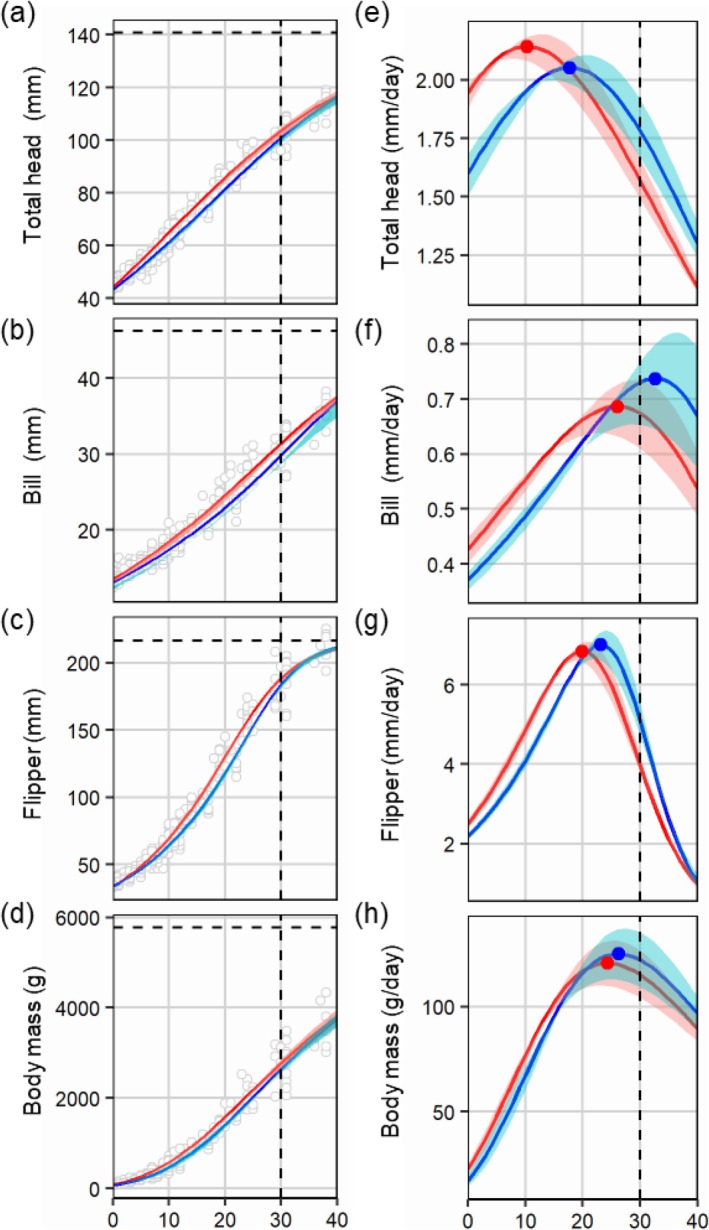
Growth curves (a–d) and daily growth rate (e–h) of first (red) and second (blue) chicks of Gentoo Penguin ( *Pygoscelis papua* ) for total head (a, e), bill (b, f), flipper (c, g), and body mass (d, h). The vertical and horizontal dashed lines represent the age at the end of the guarding period and upper asymptotes, respectively. The maximum absolute growth rates (g_max_ = *A* × *k*) at the inflection point are shown as filled circles in (e–h).

**FIGURE 4:**
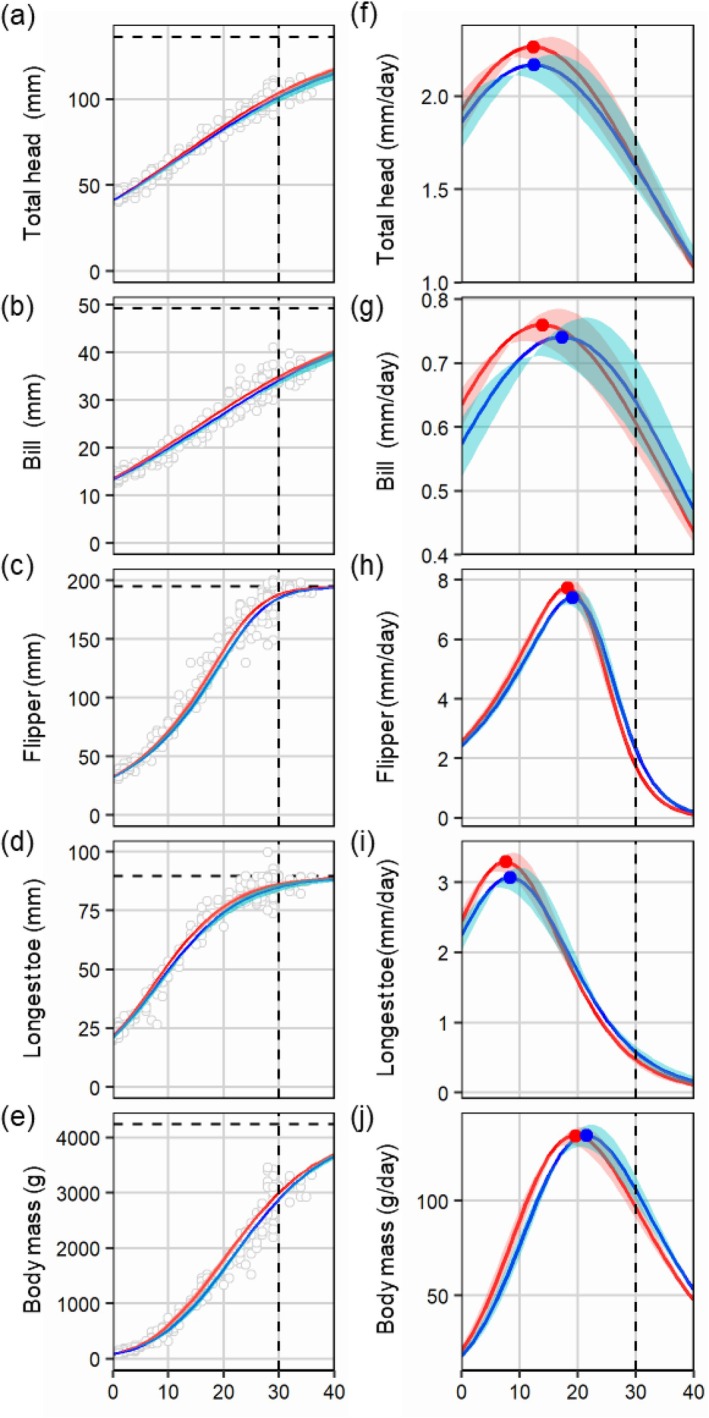
Growth curves (a–e) and daily growth rates (f–j) of first (red) and second (blue) chicks of Chinstrap Penguins ( *Pygoscelis antarcticus* ) for total head (a, f), bill (b, g), flipper (c, h), longest toe (d, i) and body mass (e, j). The vertical and horizontal dashed lines represent the age at the end of the guarding period and upper asymptotes, respectively. Maximum absolute growth rates (g_max_ = *A* × *k*) at the inflection point are shown as circles in (f–j).

We measured the growth of chicks only during the guarding period, which could cause fitted models to return a biologically meaningless low upper asymptote and a depressed regression curve (Austin et al. 2011; Ricklefs 1968b). Therefore, as suggested by Tjørve and Tjørve (2017), we used fixed asymptotes with the average values of each morphometric trait and body mass of adults of both sexes. Chinstrap and Gentoo Penguins are sexually size‐dimorphic to a subtle degree (Amat et al. 1993; Renner et al. 1998), so to avoid bias from an unbalanced sex ratio, we randomly sampled 60 individuals from each species, with an equal sex ratio, in 2014–2017. The sex of birds was determined with a molecular sexing method (Han et al. 2009). Because the same fixed asymptote was applied to both hatching order groups, the between‐group comparison of growth parameters is minimally affected by individual variation in true asymptotic size. Also, we included a random effect for individual chicks to account for the lack of independence from repeated measures for *d, k*, and *Ti*. After fitting growth models for each first and second chick group, we plotted the estimated growth curves with means and 95% confidence intervals (CIs), estimates of *d*, *k*, and *Ti* parameters, and fixed upper asymptotes (Figures 2, 3, 4). We converted the maximum relative growth rate (*k*) to an absolute maximum growth rate (g_max_) by multiplying it by the fixed upper asymptotes (g_max_ = *k* × *A*) to plot the position of the maximum growth rate (Figures 3 and 4). We removed outliers in morphometrics (values outside standard deviation × 2) due to measurement or entry error by checking the scatterplot of each trait before fitting growth curves. We estimated the absolute instantaneous daily increment in size and mass (growth rate) at a given age (*Y*_*t*_) with the following equation (first derivative; Svagelj et al. 2019) using estimated means, 95% CIs of *d*, *k*, and *Ti* parameters, and fixed upper asymptotes.

$$Y_{t}=\frac{1-d}{\left(1-d\right)\times d^{\frac{d}{1-d}}}\times A\times k\times e^{\frac{-k\left(t-\mathrm{Ti}\right)}{d^{\frac{d}{1-d}}}}\times {\left\{1+\left[\left(d+1\right)\times e^{\frac{-k\left(t-\mathrm{Ti}\right)}{d^{\frac{d}{1-d}}}}\right]\right\}}^{\frac{d}{1-d}}$$

Statistical analyses were carried out using the *nlme* package (Pinheiro et al. 2022) in R version 4.2.2 software (R Core Team 2022). To compare growth strategies between species, we qualitatively compared the estimated growth parameters (*Ti, k, d*) and asymmetry patterns between Gentoo and Chinstrap Penguins. Due to differences in sample sizes and measurement protocols between species across years, we did not conduct formal statistical comparisons between species but instead focused on describing species‐specific patterns in relation to their known life‐history differences.

### 2.4 Temporal Change in Size Asymmetry

We calculated the degree of growth asymmetry by dividing the difference in body size and mass between the first (*W*_*f*_) and second chicks (*W*_*s*_) on the same day using the field measurements.

$$\text{Asymmetry index}=\frac{W_{f}-W_{s}}{0.5\times \left(W_{f}+W_{s}\right)}$$

To examine the temporal pattern of size asymmetry, we fitted a linear mixed‐effect model for size asymmetry as a function of age and body traits/mass with the *lme4* package for R (Bates et al. 2018). We included the quadratic term of age and its interaction with body traits/mass because the asymmetry of body traits was a better fit for the quadratic line compared with a linear line in both species, based on a model comparison with a likelihood ratio test (Gentoo Penguin: χ^2^ = 58.12, *p* < 0.001; Chinstrap Penguins: χ^2^ = 70.04, *p* < 0.001). The linear mixed‐effect model also included two‐way interaction terms, including age × body traits/mass and age^2^ × body traits/mass. Nest was included as a random effect to account for repeated measurements of asymmetry by each nest. We calculated the marginal means of asymmetry up to 40 days after the first chicks hatched with the *emmeans* package for R (Russell 2020) to determine when the asymmetry peak occurred, depending on body traits/mass.

### 2.5 Accuracy of Age Estimates

We evaluated the ability of each growth model to predict the age of the chicks and determined whether the accuracy of the age estimates improved after accounting for growth differences between the first and second chicks. We estimated age (t) at a given body size and mass (*W*_*t*_) with the following equation (the inverse of the growth curve function, taking absolute values within natural logarithm functions) and mean estimates of growth curves parameters (*A, k, Ti, d*) for body mass and morphometric traits, except for the longest toe length in both species.

$$t=\left[\frac{\left\{\ln\left({\frac{W_{t}}{A}}^{1-d}-1\right)-\ln(|d-1|)\right\}\times d^{\frac{d}{1-d}}}{-k}\right]+\mathrm{Ti}$$

We conducted a 10‐fold cross‐validation to fit new growth curves for each dataset, obtain parameter estimates, and compare the accuracy of age estimates. We generated balanced random folds by hatching order (first and second chicks) and species, and used individual chicks as the resampling unit in the *groupdata2* package (Olsen 2022). For Chinstrap Penguins, we also placed data into each fold by measurement frequency (a frequent measurement group for 2013–2014 and an infrequent group for 2014–2016). Using each training dataset, we fitted three growth models each species for all individuals, first chicks, and second chicks.

We tested the effect of hatching order on age estimates by comparing the prediction accuracy of the models built with all individuals regardless of hatching order and the average predictive error of the models built for each hatching order group. We used root mean square error (RMSE) to gauge the accuracy of the age estimate. Because there is typically more variance in growth across individuals as chicks age, we also calculated the RMSE for chicks that were less than 15 days old and those older than 15 days (Lok et al. 2014).

## 3 Results

### 3.1 Gentoo Penguins

The growth curves for first and second chicks predicted that flipper length grew most closely to adult size (87.04% and 84.88%, respectively) at the end of the guarding period (30 days after hatching), followed by total head length (73.64% and 71.65%), bill length (67.97% and 64.54%), and body mass (47.94% and 45.66%, respectively) (Figure 3a–d).

Parameter estimates showed that the age at maximum relative growth rate (*Ti*) was considerably higher for second chicks than for first chicks in all skeletal measurements and body mass (Figure 2a, Table S1). The mean age at the maximum relative growth rate (*Ti*) of second chicks was 7.25 days later for total head, 6.61 days for bill length, 3.25 days for flipper length, and 1.90 days for body mass, compared with first chicks on average. The *d* parameter determining the relative size at maximum relative growth rate was lower in the first chicks than in the second chicks in total head and flipper length (Figure 2a). This indicated that the rapid growth of the second chicks occurred when they were closer to adult size, compared with the age of maximum growth of the first chicks. The maximum relative growth rates (*k*) were similar between first and second chicks in all body traits (see Figure 2a, Table S1).

In the temporal pattern of daily growth rate (Figure 3e–h), the ranking was reversed due to the later *Ti* of the second chicks compared with the first chicks (Figure 2a). Growth rates were substantially higher in first chicks than in second chicks until the mid‐guarding period (10–20 days after hatching) due to differences in *Ti*. However, the growth rate of first chicks began to decrease during the late‐guarding period, while the growth rate of morphometric traits of second chicks continuously increased and overtook those of first chicks (Figure 3e–h). Such a reversal of growth rates was notable in total head and flipper lengths. Total head and flipper lengths of second chicks at the age of maximum relative growth rate were longer than those of first chicks due to the *d* parameter (Figure 3e,g). However, even after the average growth rates for bill length and body mass of second chicks overtook those of first chicks, there was a considerable overlap in the range of daily growth rates between first and second chicks (Figure 3f,h).

**FIGURE 5:**
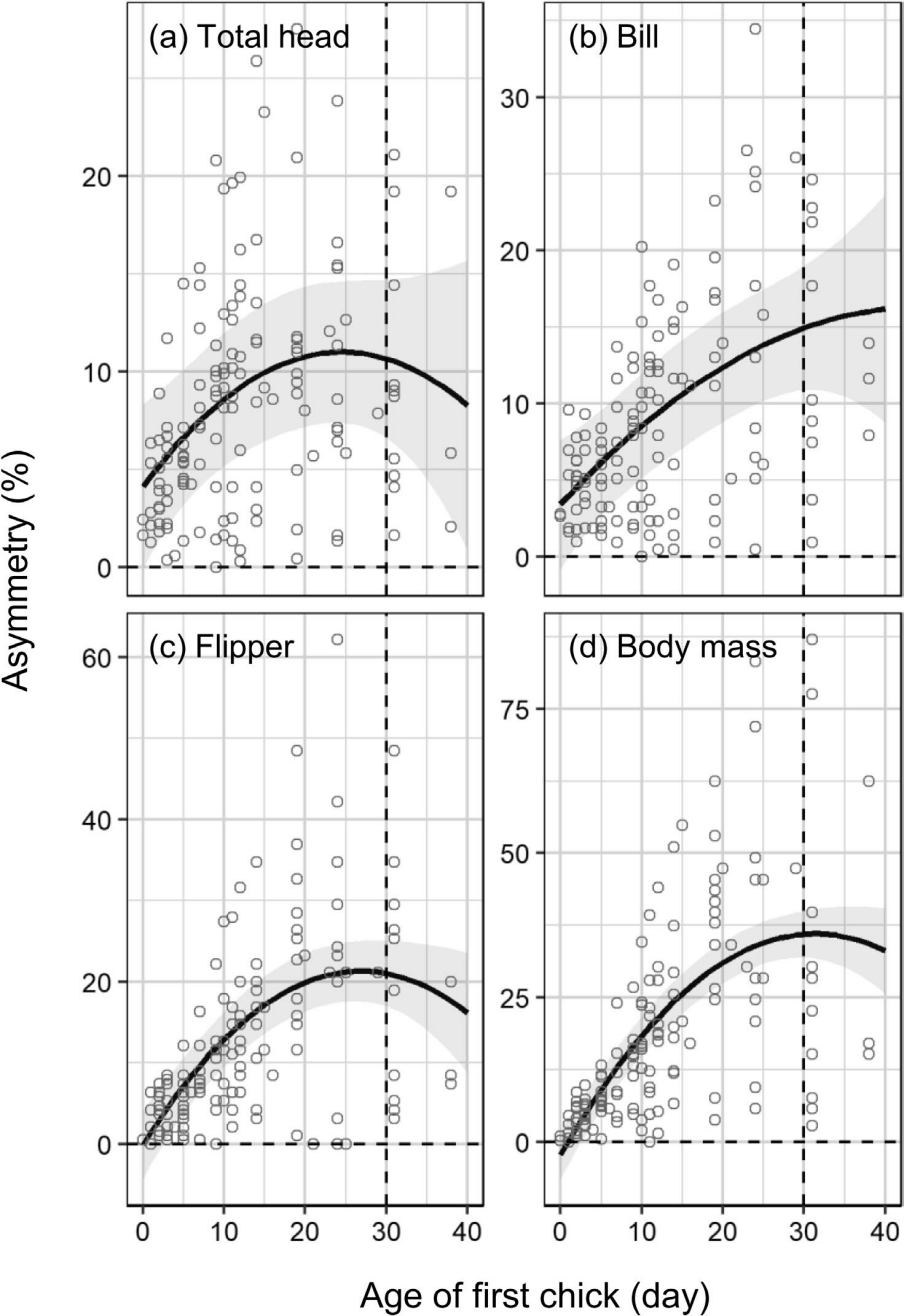
Growth asymmetry between first and second chicks for total head (a), bill (b), flipper length (c), and body mass (d) in Gentoo Penguins ( *Pygoscelis papua* ). Asymmetry is calculated as the absolute difference between siblings/mean of siblings × 100, and the absolute difference is the difference in size and mass between siblings at the same date. Solid lines and gray shaded regions represent the marginal mean and 95% confidence intervals as estimated by linear mixed‐effect models for asymmetry as a function of age and body traits.

The fitted line of size asymmetry between each brood peaked at an age of 25 days for total head, 27 days for flipper length, and 31 days for body mass (Figure 5). Asymmetry in total head and flipper length and body mass began decreasing around the late chick‐guarding period for Gentoo Penguins (Figure 5a,c,d), although the asymmetry of bill length continuously increased after the chick‐guarding period (Figure 5b).

### 3.2 Chinstrap Penguins

The fitted growth curves for first and second chicks showed that the flipper (96.49% and 94.91%, respectively) and longest toe (96.22% and 94.98%) grew to adult size at the end of guarding period, while other traits (total head = 76.06% and 74.39%; bill length = 71.01% and 69.50%) and body mass did not reach adult size (70.83% and 68.04%, respectively) (Figure 4a–e).

Growth curve models revealed that the difference in parameters between first and second chicks varied depending on the body traits (Figure 2b). For total head, flipper, and longest toe lengths, first chicks had a slightly higher *k* than second chicks, indicating that first chicks had higher maximum relative growth rates (Figure 2b). Estimates of growth rates showed that first chicks with a higher *k* grew faster than second chicks until around the age of the maximum relative growth rate (Figure 4f,h,i). However, as the growth rate of first chicks decreased more steeply, the difference in the mean rate of first chicks decreased. For flipper and longest toe lengths, the growth rates of second chicks were slightly higher than those of first chicks during the late guarding period (20–30 days after hatching; Figure 4h,i).

For body mass, the inflection point (*Ti*) of first chicks was earlier by an average of 1.89 days compared with that of second chicks (Figure 2b). The growth rate of first chicks with earlier *Ti* therefore reached a maximum rate and decreased at an earlier age compared with second chicks. This led to higher growth rates of second chicks compared with first chicks around the age of the maximum relative growth rate (Figure 4e,j). Although *Ti* for bill length was higher in second chicks than in first chicks, there was a large overlap of the 95% CIs (Figure 2b).

**FIGURE 6:**
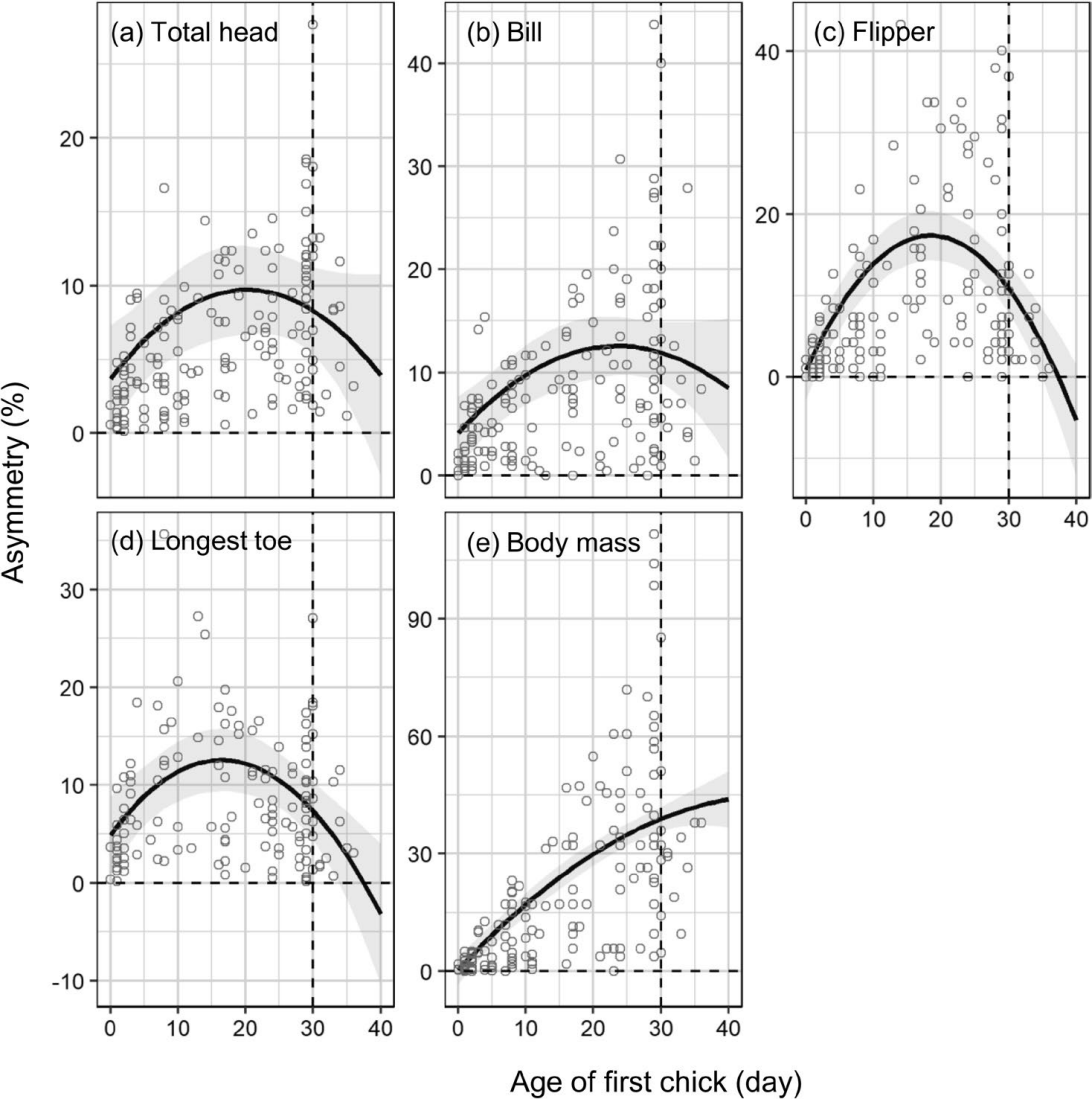
Asymmetry between first and second chick for total head (a), bill (b), flipper (c), longest toe length (d), and body mass (e) in Chinstrap Penguins ( *Pygoscelis antarcticus* ). Asymmetry is calculated as the absolute difference between siblings/mean of siblings × 100, and the absolute difference is the difference in size and mass between siblings at same date. Solid lines and gray shaded regions represent the marginal mean and 95% confidence interval estimated by linear mixed‐effect models for asymmetry as a function of age and body traits.

Growth asymmetry between first and second chicks in each brood of Chinstrap Penguins showed that the peak was earliest for longest toe length (16 days old), followed by flipper length (18 days old), total head length (20 days old), and bill length (24 days old; Figure 6). This indicates that the asymmetry for bill, total head, flipper, and longest toe metrics decreased from mid and late chick‐guarding periods. Asymmetry for the longest toe and flipper length disappeared earlier compared with other traits (Figure 6c,d). However, asymmetry for body mass continuously increased over the chick‐guarding periods (Figure 6e).

### 3.3 Age Estimation

The accuracy of age estimates from the models fitted with flipper length and body mass was higher than that of the models based on total head and bill lengths for younger chicks among Gentoo Penguins (Table 2). For older chicks, the accuracy was higher for total head and flipper lengths than for bill length and body mass (Table 2). For Chinstrap Penguins, age estimates from the model based on body mass were more accurate than those based on total head, bill, and flipper lengths, regardless of chick age (Table 2). Among skeletal measurements, the accuracy of total head length was superior to that of bill and flipper lengths (Table 2).

**TABLE 2:** Root mean square error (Mean ± Standard deviation; day) of age estimation of Gentoo ( *Pygoscelis papua* ) and Chinstrap Penguins ( *P. antarcticus* ) with data for total head, bill, flipper, and body mass.

Species	Age	Model	RMSE (mean ± SD; day) from 10‐fold cross‐validation			
			Total head	Bill	Flipper	Body mass
Gentoo Penguin	< 15 days	All individuals	1.26 ± 0.42	1.77 ± 0.50	1.08 ± 0.34	0.97 ± 0.37
		First and second chicks	1.11 ± 0.39	1.69 ± 0.49	1.04 ± 0.37	0.93 ± 0.31
	≥ 15 days	All individuals	2.34 ± 0.27	3.42 ± 0.50	2.35 ± 1.21	2.78 ± 1.02
		First and second chicks	2.12 ± 0.51	3.11 ± 0.63	2.19 ± 1.27	2.64 ± 1.11
Chinstrap Penguin	< 15 days	All individuals	1.08 ± 0.16	1.58 ± 0.29	1.61 ± 0.48	1.00 ± 0.18
		First and second chicks	1.09 ± 0.15	1.60 ± 0.33	1.85 ± 0.50	1.02 ± 0.23
	≥ 15 days	All individuals	2.58 ± 0.47	3.46 ± 0.70	11.73 ± 2.48	2.36 ± 0.46
		First and second chicks	2.53 ± 0.52	3.49 ± 0.75	9.92 ± 2.12	2.33 ± 0.44

*Note:* For the model column, “All individuals” indicates parameter estimates from the model with data from all individuals, while “first and second chicks” means parameter estimates from two models with data from first and second chicks. Mean and standard deviation of RSME results were based on 10‐fold cross‐validation.

The difference in mean RMSE depending on the growth model was less than a day in all body traits except for flipper length in Chinstrap Penguin chicks older than 15 days (Table 2). The models built for each hatching order group were 1.18 days more accurate on average for age estimates of the flippers of older Chinstrap Penguin chicks when compared with the model developed with all individual data (Table 2). However, the mean RMSE for chicks older than 15 days was greater by up to 2.85 times (body mass) for Gentoo Penguin and 7.30 times (flipper length) for Chinstrap Penguin, compared with those for younger chicks (Table 2).

## 4 Discussion

We fitted four‐parameter growth models for first and second chicks in Gentoo and Chinstrap Penguins. Regardless of hatching order, flipper length reached near the adult size at the end of the guarding period (30 days of age) in both penguin species (Figures 3 and 4). For Chinstrap Penguin chicks, longest toe length grew as rapidly as flipper length. The fastest growth in flipper length and longest toe length was consistent with previously reported findings in seabirds (Benowitz‐Fredericks et al. 2006; Gownaris and Boersma 2021; Volkman and Trivelpiece 1980), which supports the adaptive‐growth hypothesis (O'Connor 1977; Øyan and Anker‐Nilssen 1996).

Rapid development of body parts necessary for locomotion is critical for survival after the guarding period. Chicks of both species need to be able to move on land to chase parents to beg for food during the crèche period (> 30 days old) and on water for post‐crèching survival. Flippers may appear to be less important on land, as they chase their parents for food on foot, but earlier studies suggest that beginning the growth of flippers at an early growing stage may be critical because flippers must harden and widen after growing in length to be fully functional (Gownaris and Boersma 2021).

In Gentoo Penguins, growth curve models showed that second chicks exhibited delayed growth patterns relative to first chicks because they took longer to reach the age of maximum relative growth rate compared with first chicks (Figure 3). Previous studies reported that the growth rate of chicks was strongly correlated with the amount of prey provisioned by parents (Benowitz‐Fredericks et al. 2006; Braun and Hunt Jr 1983). The delayed growth of second chicks suggests that the first chicks have a competitive advantage when begging for food from parents. However, the faster growth rate of second chicks from the mid‐guarding periods and decreasing asymmetry in the later‐guarding period indicate that parents may feed more prey to second chicks. Previous studies also showed that adults of other species of penguins adjust within‐brood food allocation over time (Robertson et al. 2015; Wagner and Boersma 2019). In Magellan Penguins ( *Spheniscus magellanicus* ), prey weight relative to chick body mass was higher in second chicks during the end of the guarding period (Wagner and Boersma 2019). This could relieve the parental burden as the peak growth period does not completely overlap between the two chicks; parents can therefore allocate food to chicks that require more energy at different time periods (asynchrony) rather than at the same time. In another study, under limited food conditions, Gentoo Penguins showed higher brood reduction through the loss of second‐hatched chicks. Although second eggs are larger in mass, first‐hatched chicks gain a competitive advantage by beginning growth before their siblings hatch, ultimately outcompeting them (Williams and Croxall 1991). However, Williams and Croxall (1991) also suggest that such brood reduction and hatching asynchrony may not be directly selected for, but rather a consequence of selection for immediate incubation in cold conditions—beginning incubation upon laying the first egg to prevent chilling, which inevitably results in earlier hatching of the first chick.

We found that the difference in growth patterns between first and second chicks of Chinstrap Penguins varies by body trait (Figure 4). Total head and bill length measurements revealed that the growth rate of first and second chicks had a large overlap from the mid‐guarding periods. For flipper and longest toe length, first chicks with higher maximum relative growth rates grew faster at first, but the growth rate of second chicks overtook that of first chicks approximately 10 days after the inflection point of first chicks. We found that the age of maximum relative growth rate (*Ti*) differed considerably only for body mass, indicating that the timing of maximum growth in different body parts was less prominent between the first and second chicks of Chinstrap Penguins, unlike Gentoo Penguins (Figure 2). This suggests that first and second chicks grow almost in synchrony, which may imply a greater relative foraging burden for Chinstrap Penguin parents because they need to feed both chicks in their peak growth period.

Size asymmetry in the bill length of Gentoo Penguins continuously increased over the guarding periods, unlike those of other traits, which began to decrease before the crèching periods (Figure 5). A previous study of growth asymmetry in Chinstrap Penguin chicks found that asymmetry of flipper length and body mass largely disappeared in the late crèching periods, while that of bill length remained (Moreno et al. 1994). Bills do not reach their mature size at crèching in the *Pygoscelis* genus (Volkman and Trivelpiece 1980), and in Magellan Penguins, bill size was not a predictor of fledgling survival, unlike flipper and foot size (Koehn et al. 2016). These previous studies indicate that the growth of bills may be less critical in survival both at fledging and during the post‐fledging stage compared with flipper size during the early development stage. The later peak of asymmetry of bill length indicates that second chicks may prioritize the development of other body parts over bill length to increase post‐fledgling survival.

In Chinstrap Penguins, size asymmetry in body mass between siblings increased throughout the guarding period, while most other skeletal traits achieved reduced asymmetry near the end of the guarding period. A previous study on Magellan Penguins suggests that chicks younger than 30 days of age may allocate energy and resources to skeletal features ontogenetically, regardless of resource availability (Gownaris and Boersma 2021). In Common Murres ( *Uria aalge* ), chicks prioritize wing growth at approximately 20 days irrespective of diet and mass (Benowitz‐Fredericks et al. 2006). Such studies suggest that prioritized allocation to skeletal traits may represent an ontogenetic growth pattern in some seabird species. The delayed peak of body mass asymmetry in Chinstrap Penguins, therefore, may indicate that second chicks prioritize skeletal traits over body mass during the guarding period. Regarding skeletal traits, asymmetry in bill length began to decrease later than that of other traits in Gentoo Penguins in our study and in Chinstrap Penguins in Moreno et al. (1994). In addition, the timing of peak asymmetry and the age at which asymmetry disappeared were earliest for the longest toe length, with the fastest growth occurring during the early and mid‐guarding period. This suggests that reducing asymmetry in the longest toe length may be critical to survival after the guarding period compared with other skeletal traits.

The sequence in which asymmetry peaked and subsequently decreased generally followed the predicted order of adaptive development. In Chinstrap Penguins, asymmetry reduction occurred earliest for the longest toe (peak at 16 days), followed by the flipper (18 days), total head (20 days), and bill (24 days). This sequence aligns with the adaptive‐growth hypothesis, which prediction that body parts critical for early post‐guarding survival—specifically locomotory structures needed for terrestrial mobility (feet) and aquatic locomotion (flippers)—would show priority in reducing size disadvantages between siblings. The later reduction of asymmetry in head and bill measurements, which are less immediately critical for locomotion, further supports the notion that chicks allocate resources to minimize competitive disadvantages in functionally important structures first.

Our results revealed distinct growth strategies between Gentoo and Chinstrap Penguins that align with their contrasting life‐history traits. Gentoo Penguins showed pronounced temporal separation in peak growth timing between first and second chicks (*Ti* differences of 1.9–7.3 days depending on trait), whereas Chinstrap Penguins exhibited more synchronous growth between siblings with smaller *Ti* differences. This pattern is consistent with predictions based on their breeding phenology constraints. Chinstrap Penguins, as long‐distance migrants with later arrival at breeding colonies (Hinke et al. 2015), face a more compressed breeding window. From our observations, we suggest that the more synchronous growth pattern we observed may reflect a “time‐minimization” strategy, where both chicks must reach critical developmental thresholds (particularly flipper and foot development) within a narrow temporal window before the onset of autumn migration. This interpretation aligns with findings in other migratory species, where time‐constrained chicks exhibit accelerated or synchronized growth patterns (Reed and Clark 2016; Hirose et al. 2012). In contrast, Gentoo Penguins are short‐distance migrants or residents that can adjust breeding timing in response to local environmental conditions (Black 2016; Juarez Martínez et al. 2026). The staggered peak growth timing between siblings may represent a “risk‐spreading” strategy that distributes parental provisioning effort more evenly across the breeding season. This flexibility could buffer Gentoo Penguin populations against short‐term environmental variability, as parents can sequentially allocate resources to chicks during their respective peak growth periods rather than facing simultaneous high demand from both offspring. The contrasting asymmetry patterns in body mass further support this interpretation. In Gentoo Penguins, body mass asymmetry peaked and then decreased before the end of the guarding period, suggesting catch‐up growth by second chicks. However, in Chinstrap Penguins, body mass asymmetry continuously increased throughout the guarding period while skeletal trait asymmetry decreased. This may indicate that Chinstrap chicks prioritize skeletal development necessary for timely fledging, even at the cost of maintaining body mass asymmetry, representing a trade‐off consistent with time‐constrained breeding strategies.

There was no notable difference in the prediction accuracy of age estimates using the models built with first, second, or both chicks from the clutches of both species and across measurements (Table 2). This may be due to the relatively subtle difference between first and second chicks compared with the change in size and mass as chicks grew. Our results suggest that a growth model with all individual data was useful for estimating chick age regardless of hatching order. Cross‐validation results from age estimates showed that flipper length was more suitable for age estimates compared with total head and bill length and body mass for Gentoo Penguin chicks across all age groups within our sampling range. In contrast, for Chinstrap Penguins, the accuracy of age estimates based on total head length and body mass was higher than those based on bill and flipper lengths. This suggests that the age estimates for total head length and body mass were more reliable than those for the other two traits for Chinstrap Penguin chicks.

Our measurements were limited to the guarding period (~30 days), during which most traits had not yet reached adult size. Because freely estimating the upper asymptote from such truncated data can produce biologically unrealistic growth curves (Austin et al. 2011; Tjørve and Tjørve 2017), we used fixed asymptotes derived from adult measurements with an equal sex ratio. While this approach may not capture among‐individual variation in adult size, which could potentially influence individual‐level estimates of the relative growth rate (k), the same fixed asymptote was applied to both first and second chick groups within each species, meaning that any bias acts symmetrically and does not affect the between‐group comparisons that are central to our study. Additionally, individual‐level random effects for d, k, and Ti in our models account for a portion of such variation. Future studies with extended monitoring beyond the guarding period or incorporating sex‐specific asymptotes could further refine growth parameter estimates.

Given the sexual dimorphism observed in both species, it is possible that our results may have been confounded by sex‐based differences that were not accounted for. Recognizing this shortfall, we suggest the need for future research to include sex as an essential variable in growth analyses. We were not able to sample and identify the sexes of the chicks in this study, but at least for Chinstrap Penguins, Fargallo et al. (2006) found no evidence of a relationship between chick sex and hatching order, supporting that treatment of chick sex as a source of random error in growth models. Thus, we believe it would not introduce serious bias in our study, affecting the conclusions.

Furthermore, to gain a more comprehensive insight into these growth strategies and their consequences for fitness, future research should explore the impact of extrinsic environmental factors not considered in our study. Sauve et al. (2022) showed that growing conditions variably influenced the growth rate of chick mass depending on hatching order, with ongoing climate change likely altering these dynamics over time. Thus, future studies, analyzing how environmental variables shape growth rates across different body parts and hatching orders, and assess the subsequent effects on chick survival, could play a pivotal role in understanding seabird populations' responses to changing climate and ocean conditions.

### 4.1 In Summary

Our study found that for both species, skeletal traits essential for locomotion, such as flippers and feet, showed faster growth rates compared to other body parts. Additionally, size asymmetry between siblings decreased more gradually for bill length. These patterns suggest that chicks prioritize the development of body parts crucial for survival post‐guarding periods, potentially enhancing their fitness. Notably, the two congeneric species exhibited distinct growth strategies; Gentoo Penguins showed pronounced temporal separation in peak growth between siblings, consistent with a risk‐spreading strategy, whereas Chinstrap Penguins displayed more synchronous growth, possibly reflecting a time‐minimization strategy driven by their more constrained breeding window. For field applications, our growth models provided reliable age estimates regardless of hatching order during early postnatal growth, with flipper length being the most accurate predictor for Gentoo Penguin chicks and body mass and total head length for Chinstrap Penguins. Future studies incorporating chick sex identification and tracking chicks until they reach fully‐grown adult size would further clarify whether these growth strategies differ between male and female offspring and improve the growth curve to its full potential.

## Author Contributions

**Min‐Su Jeong:** conceptualization (lead), data curation (lead), formal analysis (lead), investigation (equal), methodology (equal), software (lead), validation (equal), visualization (lead), writing – original draft (lead), writing – review and editing (equal). **Hankyu Kim:** conceptualization (lead), data curation (equal), formal analysis (equal), methodology (lead), software (equal), validation (equal), visualization (equal), writing – original draft (lead), writing – review and editing (lead). **Woo‐Shin Lee:** conceptualization (supporting), funding acquisition (lead), investigation (supporting), project administration (supporting), resources (supporting), supervision (supporting), writing – original draft (supporting), writing – review and editing (equal). **Chang‐Yong Choi:** conceptualization (supporting), methodology (supporting), supervision (supporting), validation (equal), writing – original draft (supporting), writing – review and editing (lead).

## Funding

This study was funded by the Korean Ministry of Environment as part of a long‐term ecosystem monitoring program (via Korea Polar Research Institute; PG13130, PG14030, PG15040, PG16040, and PG17040) in the Antarctic Specially Protected Area no. 171 on King George Island. The authors thank the Korea Polar Research Institute and the King Sejong Station for their support of our field studies in Antarctica. HK was supported by grants from Kyung Hee University in 2023 (KHU‐20233274) and Korea National Research Foundation (RS‐2024‐00342882). MJ was supported by the Global—Learning & Academic Research Institution for Master’s·PhD students, and Postdocs (G‐LAMP) Program of the National Research Foundation of Korea (NRF) grant funded by the Ministry of Education (No. RS‐2025‐25442355).

## Ethics Statement

All procedures performed on live penguins were in accordance with the ethical standards and standard methods described in Commission for the Conservation of Antarctic Marine Living Resources (CCAMLR) Ecosystem Monitoring Program Standard Methods, and with permission [ILAD‐2692 (2013‐10‐18), ILAD‐3328 (2015‐11‐16), ILAD‐3960 (2016‐11‐23)] from the Korean Ministry of Foreign Affairs in accordance with the ‘Act on Antarctic Activities and Protection of Antarctic Environment’ in the Republic of Korea.

## Conflicts of Interest

The authors declare no conflicts of interest.

## Supporting information


**Table S1:** Parameter estimates (95% confidence interval in parentheses) of the growth curves for morphometric measures and body mass of the first and the second chicks of Gentoo ( *Pygoscelis papua* ) and Chinstrap Penguins ( *P. antarcticus* ).

## Data Availability

All research data and R code for fitting growth models are available in the Supporting Information.
