# Supplementary material for: Asymmetry and Asynchrony in Postnatal Growth Patterns of Two Sympatric Species of Antarctic Penguins
Source: Ecol Evol. 2026 May 14;16(5):e73563. doi: 10.1002/ece3.73563 (PMC13173763; doi:10.1002/ece3.73563)
Supplement: Supplementary file 1 — Table S1: Parameter estimates (95% confidence interval in parentheses) of the growth curves for morphometric measures and body mass of the first and the second chicks of Gentoo ( Pygoscelis papua ) and Chinstrap Penguins ( P. antarcticus ). [file ECE3-16-e73563-s001.docx]

Supplementary Table 1. Parameter estimates (95% confidence interval in parentheses) of the growth curves for morphometric measures and body mass of the first and the second chicks of Gentoo (*Pygoscelis papua*) and Chinstrap Penguins (*P. antarcticus*).

|  | *A* | *Ti* | |  | *d* | |  | *k* | |
| --- | --- | --- | --- | --- | --- | --- | --- | --- | --- |
|  |  | First | Second |  | First | Second |  | First | Second |
| Gentoo Penguin |  |  |  |  |  |  |  |  |  |
| Total head | 140.70 ± 6.08 | 10.31  (7.86–12.76) | 17.72  (14.83–20.62) |  | 1.68  (1.37–1.99) | 2.53  (2.02–3.05) |  | 0.015  (0.015–0.016) | 0.015  (0.014–0.015) |
| Bill | 46.17 ± 3.05 | 26.03  (22.93–29.13) | 32.64  (28.92–36.37) |  | 3.80  (2.96–4.65) | 5.59  (3.48–7.71) |  | 0.015  (0.014–0.016) | 0.016  (0.014–0.018) |
| Flipper | 216.37 ± 7.05 | 19.88  (18.87–20.89) | 23.13  (22.08–24.18) |  | 3.38  (2.96–3.80) | 4.41  (3.79–5.02) |  | 0.032  (0.031–0.033) | 0.032  (0.031–0.034) |
| Body mass | 5780.83 ± 614.99 | 24.30  (23.18–25.42) | 26.20  (24.97–27.44) |  | 0.97  (0.82–1.12) | 1.04  (0.90–1.18) |  | 0.021  (0.019–0.023) | 0.022  (0.020–0.024) |
| Chinstrap Penguin |  |  |  |  |  |  |  |  |  |
| Total head | 136.28 ± 5.94 | 12.30  (9.32–15.29) | 12.40  (8.77–16.04) |  | 1.96  (1.51–2.41) | 1.89  (1.38–2.40) |  | 0.017  (0.016–0.017) | 0.016  (0.016–0.016) |
| Bill | 49.18 ± 2.87 | 13.87  (10.96–16.78) | 17.26  (13.19–21.33) |  | 1.79  (1.43–2.15) | 2.13  (1.53–2.73) |  | 0.015  (0.015–0.016) | 0.015  (0.014–0.016) |
| Flipper | 194.91 ± 5.65 | 18.20  (17.29–19.10) | 19.07  (18.21–19.94) |  | 4.54  (4.09–5.00) | 4.47  (4.05–4.89) |  | 0.040  (0.039–0.041) | 0.038  (0.037–0.039) |
| Longest toe | 89.58 ± 4.24 | 7.57  (6.24–8.90) | 8.37  (6.62–10.11) |  | 1.94  (1.56–2.31) | 1.97  (1.51–2.44) |  | 0.037  (0.035–0.038) | 0.034  (0.033–0.036) |
| Body mass | 4235.00 ± 521.27 | 19.56  (18.51–20.61) | 21.45  (20.51–22.39) |  | 1.27  (1.16–1.38) | 1.39  (1.26–1.52) |  | 0.032  (0.031–0.033) | 0.032  (0.030–0.033) |
